# Resistance Training Attenuates Activation of STAT3 and Muscle Atrophy in Tumor-Bearing Mice

**DOI:** 10.3389/fonc.2022.880787

**Published:** 2022-07-01

**Authors:** Mayra Tardelli de Jesus Testa, Paola Sanches Cella, Poliana Camila Marinello, Fernando Tadeu Trevisan Frajacomo, Camila de Souza Padilha, Patricia Chimin Perandini, Felipe Arruda Moura, José Alberto Duarte, Rubens Cecchini, Flavia Alessandra Guarnier, Rafael Deminice

**Affiliations:** ^1^ Department of Physical Education, State University of Londrina, Londrina, Brazil; ^2^ Department of General Pathology, State University of Londrina, Londrina, Brazil; ^3^ Program of Molecular Carcinogenesis, Brazilian National Institute of Cancer, Rio de Janeiro, Brazil; ^4^ Department of Physical Education, State University of São Paulo (UNESP), Presidente Prudente, Brazil; ^5^ Faculty of Sport, University of Porto, CIAFEL, Porto, Portugal

**Keywords:** autophagy, cancer cachexia, muscle wasting, strength, ubiquitin-proteasome

## Abstract

**Purpose:**

Although the role of signal transducers and activators of transcription (STAT3) in cachexia due to the association of circulating IL-6 and muscle wasting has been extensively demonstrated, the effect of resistance training on STAT3 in mediating muscle atrophy in tumor-bearing mice is unknown. The aim of this study is to investigate the effects of resistance exercise training on inflammatory cytokines and oxidative-mediated STAT3 activation and muscle loss prevention in tumor-bearing mice.

**Methods:**

Male Swiss mice were inoculated with Ehrlich tumor cells and exposed or not exposed to resistance exercise protocol of ladder climbing. Skeletal muscle STAT3 protein content was measured, compared between groups, and tested for possible association with plasma interleukins and local oxidative stress markers. Components of the ubiquitin-proteasome and autophagy pathways were assessed by real-time PCR or immunoblotting.

**Results:**

Resistance training prevented STAT3 excessive activation in skeletal muscle mediated by the overabundance of plasma IL-6 and muscle oxidative stress. These mechanisms contributed to preventing the increased key genes and proteins of ubiquitin-proteasome and autophagy pathways in tumor-bearing mice, such as Atrogin-1, LC3B-II, and Beclin-1. Beyond preventing muscle atrophy, RT also prevented strength loss and impaired locomotor capacity, hallmarks of sarcopenia.

**Conclusion:**

Our results suggest that STAT3 inhibition is central in resistance exercise protective effects against cancer-induced muscle atrophy and strength loss.

## Introduction

Muscle wasting and cachexia are recognized as important and preeminent complications in cancer development and treatment (1). Cachexia, and in particular skeletal muscle wasting and weakness, leads to decreased functional capacity that negatively impacts the cancer patient’s quality of life, treatment adherence, and survival (2). Indeed, muscle wasting and cachexia development have been demonstrated to increase the length of stay (6 vs. 3 days), hospitalization cost (44%), and thereby, risk of mortality in cachectic compared to non-cachectic cancer patients (3).

Despite the relevance of cachexia syndrome to cancer patient outcomes, anti-cachexia treatments are still lacking. The currently available standard-of-care for cachectic cancer patients is limited to nutritional support, while there are still no specific drugs available to counteract muscle wasting and cancer cachexia. In this scenario, resistance exercise training (RT) emerges as an anti-cachexia strategy. Recent pre-clinical studies have demonstrated that RT counteracted muscle wasting and functional decline (4–6). However, the mechanism by which RT protects against cancer-induced muscle wasting and strength loss is still debated.

Skeletal muscle loss during cancer occurs due to an imbalance between muscle protein synthesis and degradation (7). Such imbalance has been demonstrated to be driven by the tumor that releases proteolysis induction factors and promotes a sort of modification that, in turn, causes increased reactive oxygen species (ROS) formation and chronic inflammatory state, both key triggers for proteolytic pathway up-regulation in skeletal muscle (8). Indeed, excessive ROS formation and inflammatory cytokines release (i.e., tumoral necrosis factor-alpha [TNF-α], interleukin- 6, and 1β [IL-6 and IL-1β]) are master mediators of proteolytic systems and muscle atrophy during cancer (9). Signal transducer and activator of transcription 3 (STAT3) is a member of the STAT protein family in humans that is markedly phosphorylated in response to cytokines and ROS formation, acting as a transcriptional activator of proteolytic pathways such as ubiquitin-proteasome system (UPS), the major contractile proteins degradation system in skeletal muscle (10). Recently, some studies have also indicated that STAT3 participates in the process of autophagy during cancer (11, 12). Among other related cytokines, IL-6 is a potent STAT3 activator. Indeed, pre-clinical studies have demonstrated that STAT3 is activated in muscle mediated by IL-6 excess and significantly contributes to muscle wasting during cancer (13–17). Despite the importance of STAT3 to mediate muscle wasting and the emerging anti-atrophy protective effect of RT during cancer, the effect of RT on STAT3-mediated muscle atrophy in tumor-bearing mice is unknown.

This study aimed to investigate the effects of RT on STAT3 activation by increased inflammatory cytokines and oxidative damage during tumor-induced muscle atrophy and strength loss. We hypothesized that RT may protect against the excessive activation of STAT3 by the overabundance of IL-6 and oxidative stress during cancer-induced muscle atrophy and strength loss. RT protective effects against the excessive activation of STAT3 may attenuate key targets of major proteolytic pathways, such as UPS and autophagy in skeletal muscle. Given its position downstream of a variety of cachexia-promoting factors, STAT3 would be indicated as a potential key node for future therapeutic treatments.

## Methods

### Animals and Study Design

Forty male *Swiss* mice, aged 6-7 weeks old, were obtained from our institutional animal facilities. The animals were housed in collective cages under controlled temperature (22± 10C), on a regular dark-light cycle (12h light/dark), and with free access to food (Nuvilab CR-1, Nuvital Nutrients Ltda., Curitiba, Brazil) and water during the whole experimental period. All procedures were approved by the Ethics Committee for Animal Use of the State University of Londrina (# 28336.2014.38) and followed the Guidelines of the Brazilian College of Animal Experimentation (COBEA) recommendations. Animals were placed in individual cages during the experiment to avoid confounders. Only two researchers (PSC and FTF) were responsible for group allocation at the different stages of the experiment. This study was conducted in accordance with the ARRIVE guidelines (Animal Research: Reporting of *In Vivo* Experiments).

Mice were randomly allocated in one of the following four groups: control (C, n=10), tumor-bearing (T, n= 10), exercised (E, n=10), and tumor-bearing exercised (TE, n=10). The number of animals was based on a previous study of our group (18), based on the number needed to generate cachexia (5% reduced body weight and muscle atrophy) considering an effect size of 0.90, power of 80%, and significance of 5%. There was no animal exclusion during the experiment.

Ehrlich breast carcinoma cells were inoculated two days before starting the resistance exercise training in the T and TE groups. Animals from groups C and E were injected with phosphate buffer solution (PBS). Both groups E and TE were submitted to a progressive resistance training for four weeks. Meanwhile, the physical activity of the animals in groups C and T was restricted to the space of their cages. Body weight and tumor volume were measured three times a week. The animals were euthanized 48 h after the last session of RT and fasted for 6 h; thus, the experimental design lasted 28 days of RT, 32 days of tumor growth.

### Tumor Cells Inoculation

Ehrlich breast carcinoma cells were inoculated into the right flank of T and TE mice, as previously described by Frajacomo et al. (18). Tumor cells were obtained from the ascitic intraperitoneal cavity (2.0x106 cells in 0.5ml PBS) from host animals, in a phosphate buffer (PBS, pH 7.4) with 8 μL/mL of 5,000 IU/mL heparin and centrifuged at 1,000 *g*. In a Neubauer chamber, the percentage of viable cells was then determined using trypan blue dye exclusion, in which non-viable cells were stained blue. Animals from the T and TE groups were inoculated with a suspension of Ehrlich tumor cells (1 x 106 in 100μl PBS), subcutaneously into the right flank, after which tumors were left to develop for 32 days. The inoculation of tumor cells occurred just once, and animals from the C and E groups received 100μl of PBS.

### RT Protocol

RT was performed as previously described in a ladder-climbing protocol for rats (5, 6, 19) adapted to mice. RT consisted of a set of ladder-climbing (0.5m, 0.01m grid, 900 incline) projected so it favored 8-12 dynamic movements per climb. At the top of the ladder, a dark, covered chamber was placed so the mice could rest between the climbing bouts. One week after adapting to the climbing apparatus, the mice were subjected to four to eight ladder climbs with loads attached to their tails that progressively increased in weight according to their daily performance. Thus, the maximum load achieved by each mouse in the previous training session served as a parameter to determine the subsequent training load of that mouse. The procedure was repeated three times per week for 28 days, for 12 training sessions total. Mice in the C and T-groups performed movements that were restricted to their space in their cages, except on days one and 28, when all animals were submitted to an RT protocol to determine their maximal load-carrying ability.

### Grip Strength, Locomotion, and Exploratory Activity

Once a week, the grip strength of the mice was determined using a dynamometer EEF 305 Grip Strength Meter (Insight R, Ribeirão Preto, Brazil) as previously described by Voltarelli et al. (20). The quantitative data used corresponds to the mean strength of three attempts performed by the animals. Data were reported as percentage changes from the control group. All tests were carried out under the same experimental conditions for all mice.

Locomotion and exploratory activity were determined using an open field arena (60 × 60 cm) as previously described by Voltarelli et al. (20). Briefly, the animals were recorded (digital camera, Logitech, C920, 30 Hz, fixed 90 cm above the arena) for a total of five minutes while they freely explored the open field arena. Using an automatic tracking method *via* DVideo software interface (21), the total distance traveled was measured and used as a locomotion and exploration parameter. Center crosses was calculated and used as a fragility index, since decreased exploration in the center of the open-field by the rodents is interpreted as a tendency for absence of novelty-seeking and risk-taking behavior (22).

### Euthanasia and Tissue Preparation

Forty-eight hours after the last RT session (32 days after tumor cells inoculation), the mice were anesthetized with isoflurane (5%) and euthanized by exsanguination. Blood was collected by cardiac puncture and placed into heparinized tubes, centrifuged at 1,000 *g*, and the plasma separated and stored at -80°C for further analysis. Soleus and extensor digitorum skeletal muscles (EDL) were dissected, weighed, and fixed in 4% paraformaldehyde for histological analysis. Tumors, spleen, gastrocnemius skeletal muscle, and retroperitoneal fat were also removed and weighed; gastrocnemius was quickly frozen in liquid nitrogen and then stored at -80°C for further analysis. The sum of the weights of the gastrocnemius, soleus, and EDL was used as muscle mass parameter.

### Histological Analysis

For optical microscopy analysis, soleus and EDL were fixed in 4% paraformaldehyde for 24 hours, dehydrated with graded ethanol, and embedded in paraffin blocks. Semi-thin sections of 5μm thickness were performed in a microtome, placed on glass slides, and subsequently stained with picrosirius red (H&E). For the determination of the fiber cross-sectional area (CSA) of skeletal muscles, images were captured on an optical microscope at a magnification of 100x, and the CSA of muscle fibers was quantified in six animals per group using ImageJ software (National Institutes of Health, Bethesda, MD, USA).

### Cytokines and Oxidative Stress Response Analysis

Plasma TNF-α (Ref: #88-7340-88) and IL-6 (Ref: #88-7064-88) were determined using the ELISA Ready-SET-Go kit from eBioscience (San Diego, CA, USA). Skeletal muscle samples were homogenized in 500μL PBS containing protease inhibitor. Lipid peroxidation was determined by the quantification of malondialdehyde on a thiobarbituric acid reaction substances (TBARS) as previously described by Spirlandeli et al. (23). Advanced oxidation protein products (AOPP) were determined according to Witko-Sarsat et al. (24). Reduced and oxidized glutathione levels (GSH and GSSG, respectively) were measured as described by Rahman et al. (25).

### Determination of mRNA Expression

RNA was isolated from 50 mg of frozen gastrocnemius using a RiboPure Kit (part no. AM 1924; Ambion, Austin, TX, USA) according to the manufacturer’s instructions, after which total RNA was quantified by spectrophotometry at an optical density of 260/280 (NanoDrop 2000c; Thermo Scientific, Waltham, MA, USA). An additional DNase I treatment (DNA-free kit, part no. AM1906; Ambion, Austin, TX, USA) was performed to remove contaminating genomic DNA from the isolated RNA. Next, complementary DNA (cDNA) was synthesized from 1000 ng of total RNA using a high-capacity cDNA reverse transcription kit (part no. 4374966; Applied Biosystems, Foster City, CA, USA). A quantitative polymerase chain reaction (PCR) was performed using the ViiA7 Real-Time PCR System (Applied Biosystems, Foster City, CA, USA). The following Taqman^®^ gene expression assays (Applied Biosystems, Foster City, CA, USA) were used in this study: FOXO1, FOXO3, Atrogin-1, and PGC-1α. PCR cycles were as follows: one cycle of 500C for 2 minutes, one cycle of 95°C for 20 seconds, 40 cycles of 01 second at 95°C, 20 seconds at 60°C. All amplification reactions were performed in triplicate, and peptidylprolyl isomerase A and beta-actin were used as a reference gene to normalize reactions. The relative expression was determined by the 2-ΔΔCT method.

### Immunoblotting

Proteins from gastrocnemius muscle samples were extracted using the extraction buffer [50 mM of HEPES, 40 mM of NaCl, 2 mM of EDTA, 1.5 mM of Na3VO4, 50 mM of NaF, 0.1% sodium dodecyl sulfate (SDS), 0.1% Triton X-100, and a protease and phosphatase inhibitor cocktail (#5872 Cell Signaling Technology, Danvers, MA, USA)] at 1:10 proportion. The total protein was determined by BCA assay (QPRO BCA protein assay, Cyanogen, Bologna, Italy), and equivalent amounts of 20–80 μg protein were electrophoresed on 10% SDS-PAGE in running buffer with 25 mM of Tris-base, 1.92 M of glycine (pH 8.6), and 1% SDS. Gels were blotted into a polyvinylidene difluoride (Immun-Blot^®^ PVDF Membrane, Bio-Rad, Hercules, CA, USA) in transfer buffer containing 25 mM of Tris, 192 mM of glycine (pH 8.3), and 20% methanol. Non-specific binding was blocked with 5% (w/v) dry nonfat milk in TBS-T (anti-Fbx32/atrogin-1 1:1,000 Abcam catalog #ab74023, anti-MuRF-1 1:1,000 Abcam catalog #ab172479, anti-Phospho-Stat3 (Tyr705) 1:1,000 Cell Signaling catalog #9131, anti-Stat3 1:1,000 Cell Signaling catalog #12640; Anti SQSTM1/p62 1:1,000 Cell Signaling catalog #5114; Anti-Beclin-1 1:1,100 Invitrogen Catalog #PA1-18857; Anti-LC3B 1:1,100 Invitrogen Catalog #PA5-32254) overnight at 4°C, washed, and incubated with a secondary horseradish peroxidase-conjugated anti-rabbit antibody (anti-rabbit IgG 1:4,000 Bio-Rad, Hercules, CA, USA). Immunoreactivity bands were detected by enhanced chemiluminescence ECL (GE Healthcare, Chicago, IL, USA) according to the manufacturer’s instructions. Total protein determined using a Ponceau buffer was used for normalization. Band quantification was performed by optical densitometry using Image Studio Lite Ver 5.2 (Li-Cor Biosciences, Lincoln, NE, USA).

### Statistical Analyses

All data were expressed as mean ± standard deviation except CSA that was presented in dispersion data and frequency distribution. The data normality was checked using Shapiro-Wilk and D’Agostino-Pearson tests. Two-way analysis of variance (ANOVA) was applied for parametric group comparisons. When an *F* ratio was significant, Tukey’s *post hoc* test was used to identify significant differences. The Kruskal-Wallis test compared the CSA muscles, followed by Dunn’s *post hoc* test. The Pearson’s correlation coefficient was used to determine the association among STAT3 protein content and interleukins and oxidative damage parameters. The significance level was significant when *P<* 0.05 in all cases. GraphPad Prism 5 and Origin 12.0 were used for statistical analysis and graphic production.

## Results

### Resistance Training Prevented Tumor-Induced Muscle Wasting

**Figure 1:**
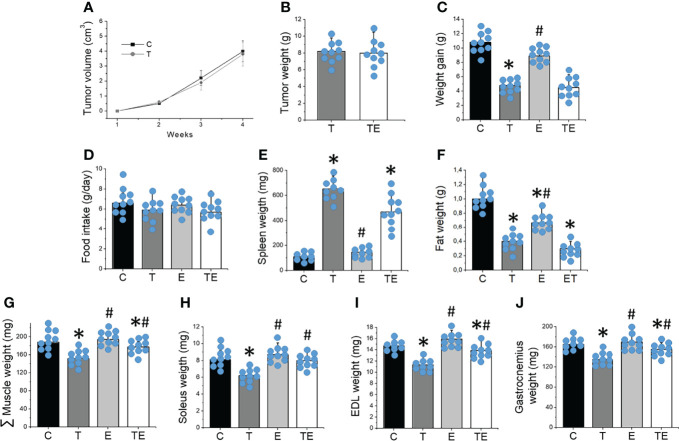
Resistance training prevented tumor-induced muscle wasting. **(A)** tumor volume over the 4 weeks (n = 10) and **(B)** tumor mass (n = 10), **(C)** weight gain (n = 10), **(D)** food intake (n = 10), **(E)** spleen and **(F)** fat weight (n = 10), **(G)** sum of skeletal muscle mass (n = 10), **(H)** soleus (n = 10), **(I)** EDL (n = 10) and **(J)** gastrocnemius (n = 10) mass after 4 weeks of experiment for groups control, tumor-bearing, exercised and tumor-bearing exercised. Results are mean ± standard deviation. *p < .05, compared with control; ^#^p < .05, compared with Tumor-bearing group (by ANOVA two-way followed by Tukey post-hoc test). C, control group; E, exercised; T, tumor-bearing; TE, tumor-bearing exercised.

Tumor grew progressively, reaching 15.5 ± 3.2% of the body weight of the mice 32 days after tumor cell inoculation in both T and TE groups. No changes in tumor volume or weight were demonstrated between T and TE groups (**Figures 1A, B**). Tumor-bearing mice exhibited significantly (*P* <.05) less weight (6.5 ± 1.1%) when compared to the controls (**Figure 1C**). Tumor-bearing mice also presented splenomegaly, significantly less retroperitoneal fat and a significant 16.1% reduction in muscle mass compared to control mice (**Figures 1E–J**). RT partially mitigated muscle wasting (*P* <.05) but did not prevent body weight and fat loss, tumor growth or splenomegaly. No changes in food intake emerged among the groups (**Figure 1D**).

### Resistance Training Attenuated Tumor-Induced Muscle Atrophy, Strength Loss, and Impaired Locomotor Capacity

**Figure 2:**
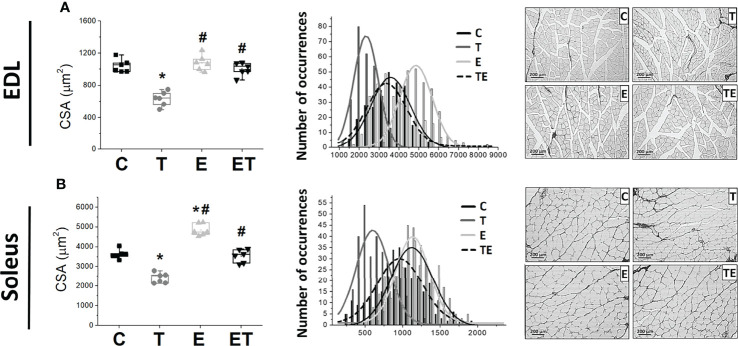
Resistance exercise attenuates tumor-induced muscle loss. Scatter plot of the median cross-sectional area (left) and cross-sectional area distribution by occurrence number (right) of **(A)** soleus muscle (n = 6) and **(B)** EDL muscles (n = 6); representative images of cross-sectional area of soleus muscle (C) in groups control, tumor bearing, exercised and tumor-bearing exercised. *p < .05, compared with control group; ^#^ *p < .05*, compared with Tumor-bearing group (by Kruskal-Wallis test followed by Dunn’s *post-hoc)*. C, control group; CSA, Cross Sectional Area; E, exercised; EDL, extensor digitorum longus; T, tumor-bearing; TE, tumor-bearing exercised.

**Figure 3:**
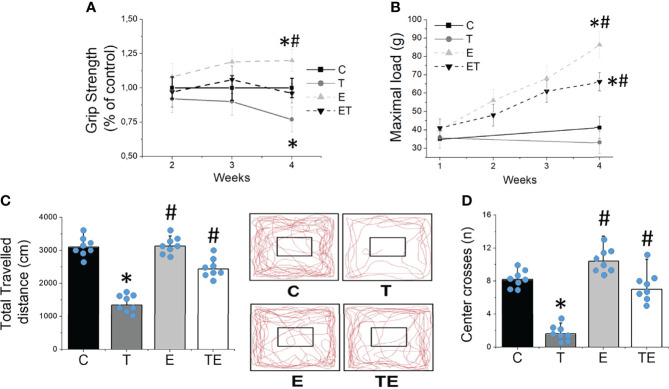
Resistance exercise preclude impaired muscle function provoked by tumor growth. **(A)** grip strength gain (n = 10) and **(B)** maximal carrying load (n = 10) over 4 weeks. **(C)** locomotor/exploratory capacity demonstrated by total travelled distance (n = 8) and **(D)** number of center crosses (n = 8) in groups control, tumor-bearing, exercised and tumor-bearing exercised. Results are mean ± standard deviation. *p < .05, compared with control; ^#^ *p < .05*, compared with Tumor-bearing group (by ANOVA two-way followed by Tukey *post-hoc* test). C, control group; E, exercised; T, tumor-bearing; TE, tumor-bearing exercised.

The tumor development provoked EDL and soleus muscle atrophy compared to the control group, as demonstrated by muscle fiber CSA (**Figure 2**). By contrast, RT mitigated the muscle atrophy in both EDL and soleus muscles (*P* <.05). Tumor development also impaired muscle strength measured by grip strength and maximal training load (**Figures 3A, B**) and locomotion and exploration capacity in tumor-bearing mice (**Figures 3C, D**). RT prevented muscle strength loss and impaired mice locomotion and exploration capacity (*P* <.05) (**Figure 3**). Notably, the maximal training load was greater in tumor-bearing exercised mice than in the healthy sedentary control group (**Figure 3B**).

### RT Attenuated Muscle Atrophy by Preventing STAT3 Phosphorylation Mediated by Decreasing IL-6 and Muscle Lipid Peroxidation

**Figure 4:**
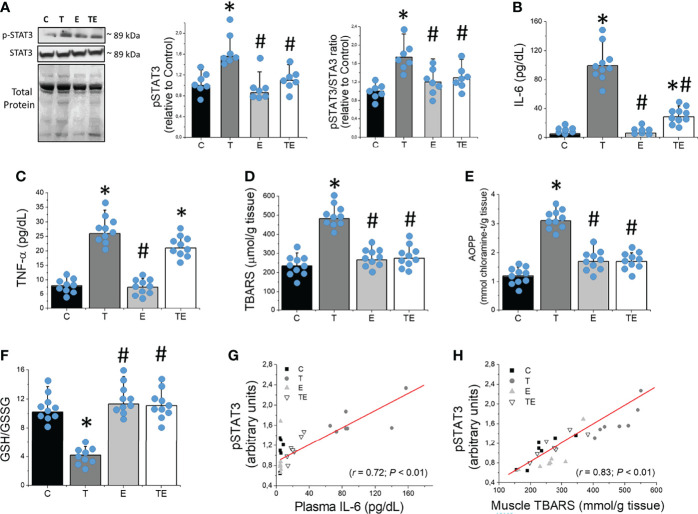
Resistance exercise prevents tumor growth-induced STAT3 phosphorylation associated to elevated IL-6 and skeletal muscle oxidative stress. **(A)** Skeletal muscle protein levels of STAT3 (n = 7), **(B)** plasmatic concentration of IL-6 (n = 10) and **(C)** TNF-α (n = 10), **(D)** skeletal muscle concentration of TBARS (n = 10), **(E)** AOPP (n = 10) and **(F)** GSH/GSSG ratio (n = 10). **(G)** Association between skeletal muscle protein levels of STAT3 and IL-6 plasma concentration and **(H)** skeletal muscle TBARS concentration (n = 7) for groups control, tumor-bearing, exercised and tumor-bearing exercised groups. Results are mean ± standard deviation. *p < .05, compared with control; ^#^ *p < .05*, compared with Tumor-bearing group (by ANOVA two-way followed by Tukey *post-hoc* test). AOPP, advanced oxidation protein products; C, control group; E, exercised; GSH, Glutathione; GSSG, Glutathione disulfide; pSTAT-3, Signal transducers of transcription- phosphorylated; STAT-3, Signal transducers of transcription; T, tumor-bearing; TBARS, Thiobarbituric acid reactive substances; TE, tumor-bearing exercised; TNF-α, tumor necrosis factor-alpha.

STAT3-phosphorylated protein content was markedly elevated in the skeletal muscle of tumor-bearing mice compared to the controls (**Figure 4A**). In agreement with previous studies using different cancer cachexia models (5, 6, 18), Ehrlich tumor development presented characteristics of pro-inflammatory scenario, as demonstrated by significant (*P* <.05) elevated TNF-α and IL-6 in plasma of tumor-bearing mice compared to the control group (**Figures 4B, C**). Tumor-bearing mice also presented skeletal muscle oxidative damage, as demonstrated by elevated levels (*P* <.05) of the lipid peroxidation evaluated by TBARS, protein oxidation evaluated by AOPP, and redox imbalance demonstrated by elevated skeletal muscle GSSG concentration and reduced GSH/GSSG ratio (**Figures 4D–F**). Notably, STAT3-phosphorylated protein content was significantly correlated with plasma IL-6 and muscle TBARS concentrations (**Figures 4G, H**). These data demonstrated that STAT3 activated in skeletal muscle is strictly associated with elevated IL-6 and lipid peroxidation and significantly contributes to muscle wasting during tumor growth. By contrast, RT prevented tumor-induced elevation on IL-6 plasma concentration and oxidative damage markers on skeletal muscle, which attenuated STAT3 phosphorylation (**Figure 4**), all events that play a key role in skeletal muscle protein degradation.

### RT Prevents the Increase of Key Genes and Proteins of Ubiquitin-Proteasome and Autophagy Pathways in Tumor-Bearing Mice

**Figure 5:**
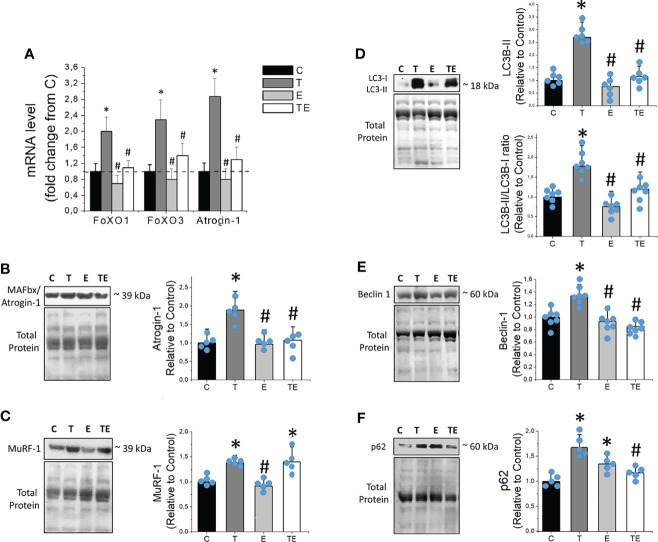
Resistance exercise prevents tumor-induced elevation of proteolytic key genes and proteins linked to ubiquitin-proteasome and autophagy system. **(A)** Skeletal muscle mRNA levels of FoXO1, FoXO3 and Atrogin-1 (n = 6). **(B)** Skeletal muscle protein levels of MAFbx/Atrogin-1(n = 5), **(C)** MuRF-1 (n = 5), **(D)** LC3B-II and LC3B-II/LC3B-I ratio (n = 6), **(E)** Beclin-1 (n = 6) and **(F)** p62, of control, tumor-bearing, exercised and tumor-bearing exercised groups. Results are mean ± standard deviation. *p < .05, compared with control; ^#^ *p < .05*, compared with Tumor-bearing group (by ANOVA two-way followed by Tukey *post-hoc* test). C, control group; E, exercised; FoXO, Forkhead box protein; LC3B-II, Light Chain 3B phosphatidylethanolamine conjugate; MAFbx, Muscle atrophy F-box; mRNA, messenger ribonucleuc acid; Murf-1, Muscle-specific RING finger protein 1; T, tumor-bearing; TE, tumor-bearing exercised.

Expression of the RNA for FoXO1, FoXO3, and Atrogin-1, key genes in skeletal muscle proteolysis, were elevated along with the tumor development (**Figure 5A**). We also observed markedly elevated expression of muscle-specific ubiquitin ligases Atrogin-1 and Murf-1 (**Figures 5B, C**), as well as autophagy pathway proteins such as LC3B-II and Beclin-1 (**Figures 5D, E**) in tumor-bearing mice compared to the control. The expression of p62 was also elevated in tumor-bearing mice compared to the control, similar to demonstrated in previous studies (26–28). Consistent with the protective effect of RT on muscle atrophy, RT prevented elevated RNA levels of FoXO1 and FoXO3, and RNA and protein levels of Atrogin-1, master regulators and a key element of skeletal muscle proteolysis on the ubiquitin-proteasome system, respectively. RT also protected skeletal muscle against tumor-induced elevated autophagy proteins LC3B-II, Beclin-1 and p62. Heathy exercised mice (E group) did not presented any changes in muscle-specific ubiquitin ligases or autophagy proteins, except for modest elevated p62 (**Figure 5F**).

## Discussion

Given the emerging data demonstrating the protective effects of RT and the importance of STAT3 on mediating muscle wasting during cachexia, we sought to determine if the protective effects of RT on tumor-induced muscle atrophy and strength loss are associated with the STAT3 signaling mechanism in skeletal muscle. Specifically, we hypothesized that STAT3 changes mediated by RT play a central role in the protective effects of RT during cancer-induced muscle atrophy and strength loss. Indeed, our results show that STAT3 activation mediated by elevated IL-6 plasma concentration and oxidative damage in skeletal muscle plays a key role in tumor-induced muscle atrophy. In contrast and remarkably, RT attenuated muscle atrophy by preventing STAT3 phosphorylation-mediated decreasing IL-6 and muscle lipid peroxidation. This protection contributed to preventing the increase of key genes and proteins of ubiquitin-proteasome and autophagy pathways in tumor-bearing mice, such as Atrogin-1, LC3B-II, Beclin-1 and p62.

Considering the invasiveness of acquiring human muscle tissue from biopsies, we used Ehrlich´s animal model to investigate the mechanisms responsible for the protective effect of exercise during cancer cachexia. We used the Ehrlich solid tumor, a model extensively used as a tool to investigate antineoplastic drugs (29, 30). We recently demonstrated that the Ehrlich tumor model reproduces functional and biological characteristics of cancer cachexia, 28 days after Ehrlich tumor cells inoculation, such as loss of body weight and muscle mass, loss of strength, and muscle atrophy (18), as our data confirmed (**Figures 1** and **2**). Regarding exercise training, ladder climbing was chosen as the RT model because it has been previously demonstrated to be an efficient model to promote muscle hypertrophy in healthy rodents (19) and in the prevention of muscle loss during cancer using different tumor models (5, 6). We also choose ladder climbing as it consists of a voluntary model of RT, the exercise suggested by a meta-analysis and some institutional consensus, to be included in the exercise routine of cancer survivors (31–33).

Chronic inflammation and ROS formation are key drivers of muscle wasting during cancer, as they are important triggers of proteolytic pathways (7). More recently, studies have demonstrated that STAT3 activation, mediated by cytokines and oxidative stress, plays a central role in regulating skeletal muscle mass during cancer progression (13–15, 17). Among inflammatory cytokines, IL-6 is a key player in STAT3 activation in skeletal muscle. Indeed, the elevated serum levels of IL-6 or IL-6 family ligands (e.g., IL-11, Leukemia Inhibitory Factor) associated with robust activation of the STAT3 pathway in skeletal muscle has been demonstrated in different cancer models in advanced stages, such as C26 (13, 15), ApcMin mice (14), and Lewis lung adenocarcinoma (17). Consistent with those, our data showed excessive activation of STAT3, demonstrated by STAT3 phosphorylation, in response to elevated IL-6 plasma concentration in Ehrlich tumor-bearing mice compared to controls. Notably, we demonstrated that STAT3 phosphorylated protein content was strictly associated with elevated IL-6 plasma concentration. This seems particularly relevant, as IL-6 is produced not only by the immune system but also directly by the tumor (34), which makes cancer a unique and severe form of muscle loss and cachexia.

Along with elevated IL-6 plasma concentration, we also demonstrated increased skeletal muscle lipid peroxidation (TBARS) and protein oxidation (AOPP) markers, combined with imbalanced skeletal muscle redox state, demonstrated by decreased GSH and GSSG ratio in tumor-bearing mice compared to control. Remarkably, TBARS concentration was significantly correlated with STAT3-phosphorylated protein content in skeletal muscle. Although some studies have already demonstrated that oxidative stress induced by hydrogen peroxide (H2O2) or ultraviolet radiation can activate STAT3 in the absence of cytokine stimulation (35, 36), the combination of both elevated inflammatory cytokine and oxidative stress may potentialize muscle wasting during tumor growth. in an *in vitro* model system of murine embryonic fibroblasts, Ng et al. (37) demonstrated that simultaneous exposure to the interleukin-6 family cytokine Leukemia Inhibitory Factor (LIF) and H2O2 drives a striking and persistent phosphorylation of STAT3, whereas STAT3 phosphorylation was only transiently increased in response to LIF alone.

Notably, our data demonstrated that RT provided protection against the activation of STAT3, overabundance of IL-6, and oxidative stress-mediated muscle atrophy in tumor-bearing mice. Although the anti-inflammation and antioxidant effects of exercise are disseminated (38, 39), our study is the first to demonstrate that the protective effects of RT against muscle atrophy during cancer are strictly related to its capacity to attenuate STAT3 phosphorylation and activation. It may seem contradictory, as resistance exercise has been demonstrated to acutely activate STAT3, a mechanism linked to muscle hypertrophy (40, 41). Guerci et al. (42) demonstrated that the IL-6-dependent activation of STAT3 is required for satellite cell proliferation in response to muscle overloading. However, the relation between resistance exercise and IL-6-dependent activation of STAT3 for skeletal atrophy/hypertrophy should consider that IL-6-dependent activation of STAT3 linked to muscle hypertrophy in response to exercise is transient (40–42), while persistent increased IL-6 and STAT3 phosphorylation and activation are linked to muscle atrophy during pro-inflammatory conditions such as cancer cachexia (13–15, 17). Indeed, cancer cachexia patients are exposed to elevated circulating IL-6 levels for weeks, while circulating IL-6 increases punctually after acute exercise. Otherwise, others accompanying our study demonstrated that exercise training inhibits STAT3 phosphorylation-mediated attenuation of pro-inflammatory condition in chronic diseases (43). Thus, when debating the role of IL-6 and reactive species-dependent STAT3 activation promoted by exercise, we should take in account that downstream target activation and its consequences for skeletal muscle might be different considering health versus disease conditions.

Beyond prevention of muscle atrophy, we demonstrated that RT was also able to prevent strength loss and impaired locomotor capacity. This is relevant given that strength loss and physical dysfunction are the hallmarks of sarcopenia, a musculoskeletal disease associated with higher mortality rates in the general population (44) and cancer survivors (45, 46). Indeed, our data demonstrated that RT-trained tumor-bearing mice presented a maximal training load greater than the healthy sedentary controls, also demonstrating that RT protects against sarcopenia. These data support the idea that RT may be part of the standard in oncology treatment, as proposed by some institutions’ positions (31, 32).

Consistent with elevated IL-6, oxidative stress, STAT3 phosphorylation, and muscle wasting, we demonstrated elevated expression of key genes and protein levels of UPS and autophagy pathways in tumor-bearing mice compared to controls. Specifically, FoXO1, FoXO3, and Atrogin-1 mRNA levels and protein Murf-1 and Atrogin-1 were markedly increased in tumor-bearing mice compared to control. The same was observed for autophagy-related proteins such as LC3B-II and Beclin-1. Indeed, STAT3 acts as a transcriptional activator of the UPS proteolytic pathway (10) and has been demonstrated to trigger autophagy during cancer (11, 12). Notably, RT was able to prevent the elevation of some important proteolytic transcriptional factors (p-STAT3, FoXO1, FoXO3) and key proteins in UPS and autophagy pathways, conferring protection against muscle wasting in tumor-bearing mice. It may seem paradoxical given that the RT is an exercise modality known to promote hypertrophy and muscle growth. Therefore, studies from our group have demonstrated that in healthy rats, RT promotes the activation of mTORC1 signaling by increasing the phosphorylation of p70S6K, which is associated with skeletal muscle hypertrophy (6). In tumor-bearing rats, however, the RT-induced attenuation of myofiber atrophy was independent of the activation of anabolic mTORC1 activation; RT prevented muscle atrophy during cancer, which was associated with reduced inflammation, oxidative damage, and UPS proteins expression (6). Coherently, White et al. (47) demonstrated that IL-6 signaling inhibition after the initiation of cancer cachexia suppresses the progression of cachexia by sparing muscle mass independently of changes in muscle protein synthesis. These results suggest that proteolysis inhibition is central in the protective effects of RT against cancer-induced muscle atrophy and strength loss. In addition, beyond the ability to confer intrinsic local skeletal muscle protective phenotype, RT is also able to confer a systemic organic response against tumor growth and the released muscle proteolytic factors such as inflammatory interleukins (5, 48), mechanisms that contribute to protection against cancer-induced muscle wasting.

Autophagy operates a massive amount of proteolysis in different tissues, including skeletal muscle (12). Indeed, altered lysosomal function has also been reported in several myopathies (47), including muscle wasting and cachexia in cancer patients (49) and in pre-clinical models (26–28). The role of STAT3 on autophagy activation is less known. Studies have proposed divergent effects of STAT3 phosphorylation on autophagy; reports have indicated that phosphorylation of STAT3 promotes autophagy activation (50), while others have indicated that p-STAT3 has an inhibitory effect on autophagy flux (12, 51). Our study demonstrated that STAT3 phosphorylation coincides with enhanced autophagy, demonstrated by increased protein expression of LC3B-II and Beclin-1. Indeed, both the lipidated form of microtubule-associated protein 1 light chain 3B (LC3B-II) and the ratio between II and I LC3B isoforms, which are considered reliable markers of autophagosome formation, are significantly elevated in the muscle of tumor-bearing mice compared to controls. In the same way, Beclin-1, a main upstream regulator of autophagic sequestration, was also elevated in tumor-bearing mice compared to controls. Important to note moreover, that increased p62 was also demonstrated in tumor-bearing mice compared to controls, results that, at first glance, contrast with the observation that autophagy is clearly enhanced. These results however, are compatible with previously studies using different cancer cachexia models (26–28). Indeed, p62 accumulation can be the result of either increased autophagic sequestration or reduced autophagosome clearance (52). Penna et al. (26) demonstrated elevated skeletal muscle LC3-II after colchicine administration – a microtubule-destabilizing agent that interacts with tubulin – demonstrating that autophagy is activated in the muscle of cancer cachectic animals even with elevated p62. Notably, we demonstrated that RT inhibited STAT3 phosphorylation, which was accompanied by the prevention of increased autophagic activation. Therefore, our study only suggests, in the absence of an autophagic flux experiment, that the phosphorylation of STAT3 in response to exacerbated IL-6 and ROS formation seems to be associated with autophagy activation during cancer cachexia. In addition, key autophagy protein inhibition seems to play an important role in the protective effects of RT on tumor-induced muscle atrophy since it is directly associated with persistent IL-6 production and phosphorylation of STAT3.

Of note, heathy exercised mice presented elevated p62 compared to controls, despite no changes in LC3B-II and Beclin-1, which, on the surface, seems paradoxal given that the levels of p62 binds LC3 and substrates marked for degradation by ubiquitylation. However, the effects of resistance training on p62 levels in muscle of healthy rodents and humans appear to be controversial. In fact, studies have shown elevated (53), reduced (54, 55) or unchanged (27, 56) p62 muscle levels promoted by RT. That controversy may be explained due to the multifunctional role of p62, protein involved in many signal transduction pathways, including nutrition sensing (*via* mTORC1), inflammation and apoptosis (*via* NF-κB), antioxidant response (*via* Nrf2) (57), in addition to autophagy regulation. In fact, studies have demonstrated p62 and Nrf2 are essential for exercise-mediated enhancement of antioxidant protein expression in skeletal muscle (58). Yamada et al. (58) demonstrated Nrf2 translocation into nuclei is a key event in exercise-mediated increase in antioxidant capacity of skeletal muscle; exercise-induced Nrf2 nuclei translocation can enhance the expression of p62 at the transcription level by directly binding to the promoter region of *p62* gene, forming a positive feedback loop. These authors also demonstrated that the loss of p62 in muscle significantly reduced regular exercise-mediated increase of antioxidant enzyme expression (*i.e*., CuZnSOD and EcSOD), mimicking observations in Nrf2 mKO mice. Thus, we speculate that the slight increase in p62 without changes in the LC3-II/I ratio we found in heathy mice is compatible with the multifunctional action on this protein, such as exercise induced antioxidant activity. This however, must be further investigated in future studies.

It is important to mention that STAT3 is a transcriptional factor demonstrated to (co-)activate various metabolic pathways beyond the FoXO superfamily, UPS, and autophagy in skeletal muscles. Indeed, studies have demonstrated a vast diversity of assigned functions and other tissues not examined in our study, including tumors (59). In addition, studies have demonstrated that acute phosphorylation level of STAT3 imposed by IL-6 addition (12) or physical exercise (40, 41) is associated with muscle growth and may cause inhibited UPS and autophagy signaling (12). This response differs from that demonstrated during persistent inflammation, as the model we used in the present study. Thus, the STAT3 pathway’s complexity warrants attention when considering its roles in RT-induced protection against cancer-induced muscle atrophy. Other pathways, functions, and targets of STAT3 should be considered in the future. It is also important to mention that some limitations must be considered in the present research. The absence of additional experiments using STAT3-modulating agents, as well as the absence of autophagy flux assessment can be considered limitations of the present study. These could bring new and important data about the role of STAT3 and autophagy on cancer cachexia and RT-protection effects.

In conclusion, our data demonstrated that RT prevents against STAT3 excessive activation in skeletal muscle mediated by the overabundance of plasma IL-6 and muscle oxidative stress, a key role to attenuate cancer-induced muscle atrophy. These mechanisms contributed to preventing the increase of key genes and proteins of ubiquitin-proteasome and autophagy pathways in tumor-bearing mice, such as Atrogin-1, LC3B-II, and Beclin-1. Beyond preventing muscle atrophy, RT also prevented strength loss and impaired locomotor capacity, conditions associated with higher mortality rates and better life quality in cancer patients.

## Data Availability Statement

The raw data supporting the conclusions of this article will be made available by the authors, without undue reservation.

## Ethics Statement

The animal study was reviewed and approved by Ethics Committee for Animal Use of the State University of Londrina (# 28336.2014.38) and followed the Guidelines of the Brazilian College of Animal Experimentation (COBEA) recommendations.

## Author Contributions

MT designed the study, participated in data collection, contributed to analysis and interpretation of data and wrote the final version of the manuscript. PC participated in data collection, contributed to analysis and interpretation of data and wrote the final version of the manuscript. PM participated in data collection, contributed to analysis and interpretation of data. FF designed the study, participated in data collection, contributed to analysis and interpretation of data and wrote the final version of the manuscript. CP participated in data collection, contributed to analysis and interpretation of data and assisted in the preparation of the manuscript. PP contributed to analysis and interpretation of data and assisted in the preparation of the manuscript. JD designed the study, contributed to analysis and interpretation of data and wrote the final version of the manuscript. RC provided specialized technical assistance to the project. FG contributed to analysis and interpretation of data and assisted in the preparation of the manuscript. RD designed the study and wrote the final version of the manuscript. All authors contributed to the article and approved the submitted version.

## Funding

This study was supported by Coordenação de Aperfeiçoamento de Pessoal de Niível Superior – Brazil (CAPES) and Fundação Araucaíria. RD is supported by CNPq-Brazil #306842/2021-1 and #403232-2021-0.

## Conflict of Interest

The authors declare that the research was conducted in the absence of any commercial or financial relationships that could be construed as a potential conflict of interest.

## Publisher’s Note

All claims expressed in this article are solely those of the authors and do not necessarily represent those of their affiliated organizations, or those of the publisher, the editors and the reviewers. Any product that may be evaluated in this article, or claim that may be made by its manufacturer, is not guaranteed or endorsed by the publisher.
